# Radiation Exposure to the Thyroid After the Chernobyl Accident

**DOI:** 10.3389/fendo.2020.569041

**Published:** 2021-01-05

**Authors:** Vladimir Drozdovitch

**Affiliations:** Division of Cancer Epidemiology and Genetics, National Cancer Institute, NIH, DHHS, Bethesda, MD, United States

**Keywords:** Chernobyl, thyroid, radiation, exposure, Iodine-131

## Abstract

**Introduction:**

The Chernobyl accident resulted in a considerable release of radioactivity to the atmosphere, particularly of Iodine-131 (^131^I), with the greatest contamination occurring in Belarus, Ukraine, and western part of Russia.

**Material and Methods:**

Increase in thyroid cancer and other thyroid diseases incidence in population exposed to Chernobyl fallout in these counties was the major health effect of the accident. Therefore, a lot of attention was paid to the thyroid doses, mainly, the ^131^I intake during two months after the accident. This paper reviews thyroid doses, both the individual for the subjects of radiation epidemiological studies and population-average doses. Exposure to ^131^I intake and other exposure pathways to population of affected regions and the Chernobyl cleanup workers (liquidators) are considered.

**Results:**

Individual thyroid doses due to ^131^I intake varied up to 42 Gy and depended on the age of the person, the region where a person was exposed, and their cow’s milk consumption habits. Population-average thyroid doses among children of youngest age reached up to 0.75 Gy in the most contaminated area, the Gomel Oblast, in Belarus. Intake of ^131^I was the main pathway of exposure to the thyroid gland; its mean contribution to the thyroid dose in affected regions was more than 90%. The mean thyroid dose from inhalation of ^131^I for early Chernobyl cleanup workers was estimated to be 0.18 Gy. Individual thyroid doses due to different exposure pathways varied among 1,137 cleanup workers included in the epidemiological studies up to 9 Gy. Uncertainties associated with dose estimates, in terms of mean geometric standard deviation of individual stochastic doses, varied in range from 1.6 for doses based on individual-radiation measurements to 2.6 for “modelled” doses.

**Conclusion:**

The ^131^I was the most radiologically important radionuclide that resulted in radiation exposure to the thyroid gland and cause an increase in the of rate of thyroid cancer and other thyroid diseases in population exposed after the Chernobyl accident.

## Introduction

The Chernobyl accident that occurred on 26 April 1986 led to widescale radioactive contamination of territories in Belarus, Ukraine and the western part of Russia. The accident resulted in the release from the damaged reactor of a large amount of radionuclides into the atmosphere, including the radiologically significant short-lived Iodine-131 (^131^I), Tellurium-132 (^132^Te), Iodine-133 (^133^I), and long-lived Caesium-134 and Caesium-137 (^134^Cs and ^137^Cs) (1). Two main groups of people were exposed to radioactive fallout: (1) representatives of the population in the contaminated territories in Belarus, Ukraine, and Russia; and (2) cleanup workers (emergency and recovery workers or liquidators) who were the first responders or participated in cleanup activities at the site of the Chernobyl nuclear power plant (NPP) and in the 30-km zone around the NPP. Young children were among the population groups most affected by accident as they consumed cow’s milk contaminated with ^131^I.

The increase of thyroid cancer among persons who were exposed to Chernobyl fallout during childhood and adolescence were reported a few years after the accident, first in Belarus (2) and in Ukraine (3), and later in Russia (4). A number of radiation epidemiology studies have demonstrated an increased risk of thyroid cancer and other thyroid diseases associated with exposure of the thyroid gland to ^131^I (5–12). Results from these and other studies conducted in the affected population (13–15) suggested that an increase in the incidence of thyroid cancer in individuals exposed in childhood and adolescence was the main health effect of the Chernobyl accident. The excess odds ratios (EOR) of radiation-related thyroid cancer derived in these cohort and case-control studies were similar within the range of uncertainties and varied from 1.36 Gy^-1^ (95% confidence interval (CI): 0.39–4.15) (10) to 8.4 Gy^-1^ (95% CI: 4.1–17.3) (14). An increased risk of thyroid cancer, not statistically significant, (EOR/Gy = 3.91, 95% CI: –1.49, 65.7) has been reported in individuals exposed *in utero* (16, 17). Studies among Chernobyl cleanup workers, who were exposed as adults, also reported an increased risk of thyroid cancer after exposure to external irradiation and internal irradiation from ^131^I intake (18–20).

Since the main health effect of the Chernobyl accident is an increase in the incidence of thyroid cancer and other thyroid diseases, a lot of efforts have been devoted to the assessment of radiation thyroid doses. The main purpose of this paper is to summarize the methods and results of reconstruction of radiation doses to the thyroid of the population exposed to the Chernobyl accident.

## Thyroid Doses to The Members of The General Public

In the aftermath of the Chernobyl accident, the radiation absorbed dose to the thyroid gland for the members of the general public resulted mainly from intake of ^131^I. In brief, the radionuclides, which were released into atmosphere during the accident, deposited on the ground surface, and contaminated the pasture grass and leafy vegetables covering the ground. The grazing cows ate contaminated grass and some fraction of the radioactivity was transferred to their milk. The consumption of fresh cow’s milk contaminated with ^131^I was the main pathway of thyroid exposure while the ^131^I intake with leafy vegetables and inhaled contaminated air playing a minor role. The radiation dose due to ^131^I intake is the highest for the thyroid gland as iodine accumulates in this organ. Thyroid doses in children are higher than that of adults is because of the smaller size of the thyroid gland in children. Since the half-life of ^131^I is 8.02 days, radiation exposure to the thyroid gland occurred during the first two months after the accident when the activity of ^131^I in the environment became negligible.

In addition, there were other contributors to the thyroid exposure, which were typically rather small for most individuals, but relatively important for those with no or little milk consumption: (1) internal irradiation due to intake of short-lived radioiodine and radiotellurium isotopes (^132^I, ^133^I, ^135^I, ^131m^Te, and ^132^Te); (2) external irradiation from gamma-emitted radionuclides deposited on the ground; and (3) internal irradiation resulting from intake of long-lived ^134^Cs and ^137^Cs.

There are two types of doses to the members of general public: (1) an individual dose for a specified person that takes into account (i) information on individual whereabouts and consumption history collected, typically, by means of personal interview and (ii) individual-based radiation measurements, if available; and (2) a population-average dose for an unspecified individual that is estimated using generic values of dosimetry models. Estimates of individual dose are required for radiation epidemiological studies while the population-average doses are used for the radiation protection of population by comparing of exposure levels in population groups of different ages living in different territories.

Radiation thyroid doses are also classified as “instrumental” doses, which are estimated using individual-based radiation measurements, and ‘modelled’ doses, which are estimated using dosimetry models.

### Radiation and Thyroid Volume Measurements Available for Exposure Assessment

The following information was available to reconstruct radiation doses to the population in Belarus, Ukraine and Russia:

– About 400,000 measurements of ^131^I thyroidal activity that were derived from gamma-spectrometric and radiometric measurements (called “direct thyroid measurements”) made between 26 April and 30 June 1986 among the population resided in contaminated areas (21–23). These measurements were used to estimate the thyroid doses due to ^131^I intake for measured individuals.
– ^137^Cs ground deposition density measured in almost every contaminated settlement in Belarus, Ukraine and Russia (24).
– Ground deposition densities of ^131^I and other gamma-emitting radionuclides (^95^Zr, ^95^Nb, ^103^Ru, ^106^Ru, ^134^Cs, ^141^Ce, ^144^Ce), measured in some locations (25–28).
– Activity concentration of ^131^I and total beta-activity in cow’s milk (29–31).
– More than 600,000 measurements of radiocesium activity in individuals living in contaminated territories made using whole-body counter (32–35);
– Thyroid volume-values measured by the Sasakawa Memorial Health Foundation in Belarus, Ukraine, and Russia in the 1990s (36–38).

### Thyroid Doses from Intake of ^131^I

#### Individual Thyroid Doses for the Subjects of Epidemiological Studies

**Figure 1:**
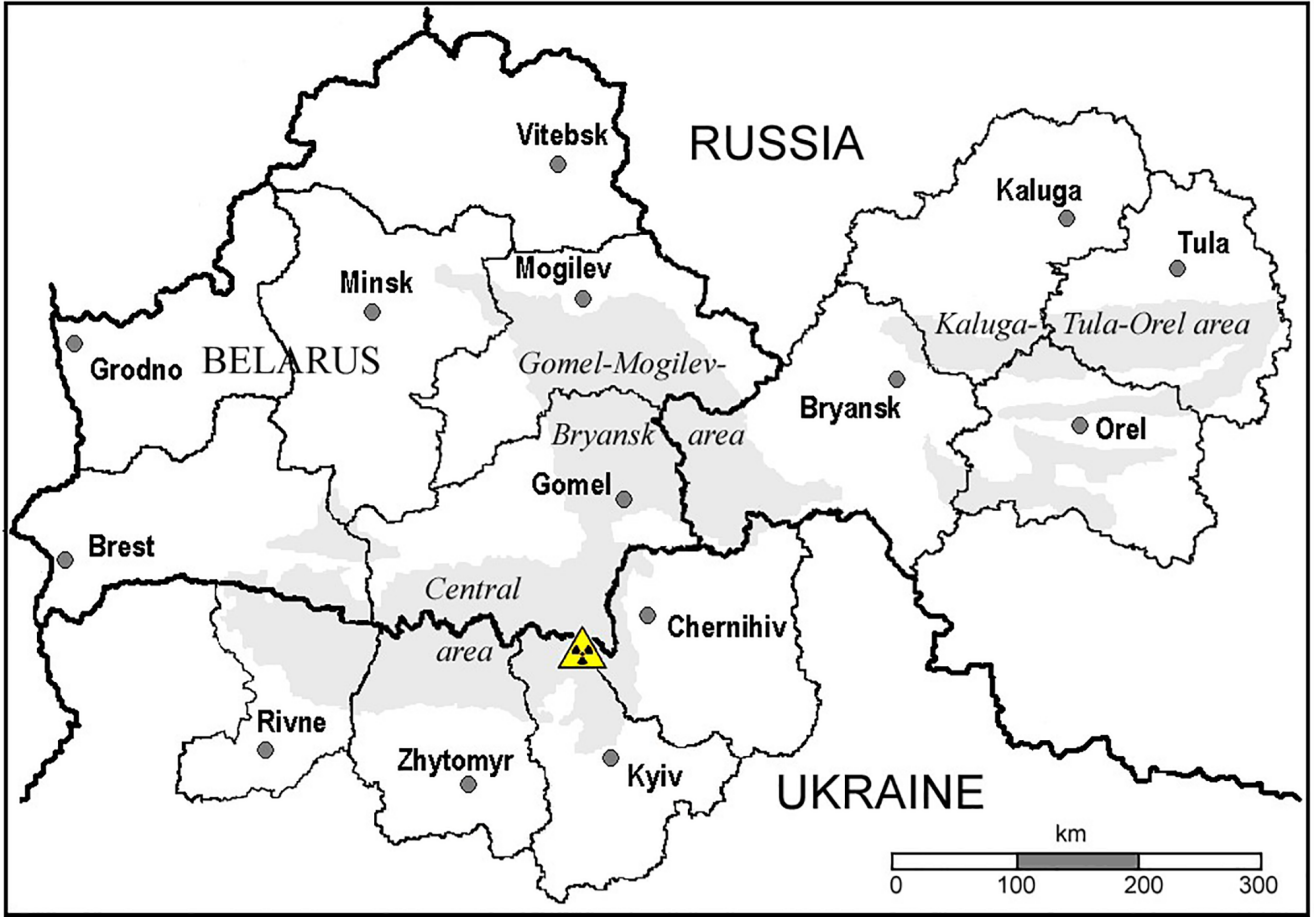
Areas in Belarus, Ukraine, and Russia. Territories with ^137^Cs ground deposition density greater than 37 kBq m^-2^ are shown in gray.

The most reliable individual thyroid doses were estimated based on the results of measurements of ^131^I thyroidal activity carried out in the most contaminated oblasts (regions) in Belarus, Ukraine, and Russia (**Figure 1**). There are two cohort studies that used estimates of individual ^131^I thyroidal activity available for each cohort member and provided the uncertainties associated with estimates of thyroid doses due to ^131^I intake. These two thyroid screening cohorts consist of people who were 0–18 years old at the time of the accident (ATA), 11,732 individuals in the Belarusian-American (BelAm) cohort and 13,204 individuals in the Ukrainian-American (UkrAm) cohort (39). The BelAm cohort includes persons who resided ATA in Gomel and Mogilev Oblasts as well as in the city of Minsk; UkrAm cohort consists of residents of Chernihiv, Kyiv, and Zhytomyr Oblasts (see **Figure 1**).

Information on residential history, consumption of cows’ milk, dairy products, and leafy vegetables as well as the administration of stable iodine necessary for the assessment of the individual thyroid doses was collected for each cohort member by means of personal interviews (40, 41). Another two screening thyroid cohorts consist of persons exposed *in utero*, 2,965 and 2,582 individuals in Belarus and Ukraine, respectively (16, 42). A fraction of the cohorts’ members, around 10% in Belarus and 28% in Ukraine, was subject to direct thyroid measurements (43, 44). Practically the same methodology was used to assess the individual thyroid doses in the BelAm and UkrAm cohorts as well as in the Belarusian and Ukrainian *in utero* cohorts.

In the BelAm and UkrAm studies, thyroid doses due to ^131^I intake were calculated in a stochastic mode using a Monte-Carlo simulation procedure that provides an estimate of the uncertainties (41, 45). In accordance with this procedure, 1,000 *individual stochastic* thyroid doses were calculated, considering the classification of errors as shared or unshared. The distribution of *individual stochastic* thyroid doses for cohort members was approximately lognormal; the geometric standard deviation (GSD) of this distribution characterized the uncertainty of dose estimates.

**Table 1** shows the distribution of individual thyroid doses in these four cohorts. More than 2/3 of the subjects of the BelAm and UkrAm cohorts received thyroid doses less than 0.5 Gray (Gy) and of the Belarusian and Ukrainian *in utero* cohorts received thyroid doses less than 0.05 Gy. Individual thyroid doses varied widely from 0 to 39, 42, 15, and 3.2 Gy in the BelAm, UkrAm, Belarusian and Ukrainian *in utero* cohorts, respectively.

**Table 1:** Distribution (%) of thyroid doses from intake of ^131^I and mean, median, and maximal dose among subjects of the screening cohorts in Belarus and Ukraine (41, 43–45).

Thyroid dose (Gy)	Belarusian cohort^a^	Ukrainian cohort^a^	Belarusian *in utero* cohort^b^	Ukrainian *in utero* cohort^b^
<0.05	16.9	18.1	67.3	73.9
0.05–0.199	25.6	32.9	18.4	17.0
0.2–0.499	24.0	21.4	9.2	6.2
0.5–0.99	15.9	12.3	3.3	1.9
1.0–4.99	15.9	13.4	1.6	1.0
5–9.99	1.3	1.3	0.2	–
≥10	0.4	0.6	0.03	–
Arithmetic mean	0.68	0.65	0.12	0.072
Median dose	0.27	0.19	0.014	0.012
Maximal dose	39	42	15	3.2
N of persons	11,732	13,204	2,965	2,582

^a^Distribution is shown for arithmetic mean of individual stochastic doses.

^b^Prenatal thyroid dose.

The *individual stochastic* doses were characterized by the GSDs from 1.3 to 5.1 with (an arithmetic mean (AM) of 1.8, a geometric mean (GM) of 1.7) for the BelAm cohort, and from 1.3 to 10.6 (AM of 1.6, GM of 1.5) for the UkrAm cohort. The uncertainties in thyroid doses were mainly defined by sources of unshared (classical) errors: the derivation of ^131^I activity in the cohort member’s thyroid from direct thyroid measurements and the values of thyroid mass (41, 45, 46).

If the result of measurement of ^131^I thyroidal activity was not available for the individual, thyroid doses were estimated using two types of models:

– Purely empirical models that were based on the correlation between environmental contamination (deposition density of ^131^I or ^137^Cs, ^131^I activity concentration in cow’s milk) and thyroid doses derived from direct thyroid measurements done among individuals of different ages (21, 22, 29, 47–49); and
– An environmental transfer model that considers a process of ^131^I activity transfer to the human thyroid with contaminated milk and/or leafy vegetables for ingestion or with contaminated ground-level air for inhalation (50, 51).

To estimate the individual modelled thyroid doses, a personal interview was conducted to collect information on the whereabouts and consumption history for a given person. Individual thyroid doses estimated using the models, called “modelled” doses, were associated with uncertainties arose mainly from the estimates of ^131^I ground deposition density in the place of residence, the transfer of ^131^I to cow’s milk, and estimates of the thyroid mass (46, 47, 52).

**Table 2** presents the thyroid doses due to ^131^I by age and by country of residence among the subjects of the case-control study of thyroid cancer in Belarus and Russia (**Figure 1**), which was coordinated by the International Agency for Research on Cancer (IARC). Data from the table show that the thyroid dose decreased with increasing age. The thyroid dose in Russia was estimated to be more than five times lower than that in Belarus, 0.10 *vs.* 0.54 Gy for mean. The *individual stochastic* doses were characterized by the GSDs from 1.59 to 3.61 (AM of 1.94, GM of 1.89). The uncertainties in thyroid doses were defined by the shared (Berkson) errors in parameters of the model and by unshared (classical) errors related to the estimates of thyroid-mass values (52, 53).

**Table 2:** Thyroid doses due to ^131^I intake at different ages and by country of residence among subjects of the case-control study of thyroid cancer in Belarus and Russia (53).

Age (year)	Thyroid dose^a^ (Gy)	
	Belarus	Russia
<2	0.70	0.43
2–4.9	0.51	0.14
5–9.9	0.38	0.033
10–14.9	0.19	0.021
15–18	0.20	0.020
Arithmetic mean (Gy)	0.54	0.10
Median dose (Gy)	0.29	0.022
Maximal dose (Gy)	8.7	4.9
N of persons	1,695	534

^a^Arithmetic mean of individual stochastic doses.

#### Population Average Dose Estimates

Thyroid doses from intake of ^131^I for population groups were estimated using a combination of the methods indicated above. The following groups of population were considered (54, 55): evacuees from the 30-km zone around Chernobyl NPP and residents of the contaminated areas in the most affected countries, Belarus, Ukraine and Russia.

*Evacuees*. More than 100,000 persons were evacuated in the weeks after the accident from the most contaminated 30-km zone around the Chernobyl NPP in Ukraine and Belarus. The thyroid doses varied with place of residence, date of evacuation, and the age of the evacuees. Evacuees from Belarusian villages received the highest doses, the average thyroid dose was estimated to be 0.68 Gy for adults and 3.1 Gy for young children (0–7 years old) *vs.* 0.28 Gy and 1.2, respectively, for evacuees from Ukrainian villages (54). The thyroid doses for the residents evacuated from the town of Pripyat were 0.28 Gy for adults and 0.99 Gy for young children received mainly due to the ^131^I intake with cow’s milk during their stay in the villages where they were evacuated (56). The population-weighted average thyroid dose for the entire evacuated population was 0.47 Gy. It should be noted that dose estimates were based on direct thyroid measurements carried out among evacuated persons.

*Residents of the Contaminated Areas*. **Table 3** shows estimates of thyroid doses due to ^131^I for the populations of Belarus, Ukraine, and Russia (1, 57). Population-average thyroid doses depended on the varied from region to region dates of fallout and ^131^I ground deposition densities, and on the dates when pasture grazing season started. The latter is vital, since cow’s milk was the main source of ^131^I intake to the exposed population. The highest Oblast-average thyroid dose due to ^131^I intake among the three countries was realized in the most contaminated Gomel Oblast in Belarus, it varied from 0.15 Gy for adults to 0.75 Gy for young children. The highest dose in Ukraine was found in Zhytomyr Oblast, 0.06 Gy for adults and 0.23 Gy for children 0–7 years old; and in Russia in Bryansk Oblast, 0.026 Gy for adults and 0.16 Gy for children 0–7 years old.

**Table 3:** Estimates of thyroid doses due to ^131^I intake for the populations of Belarus, Ukraine, and Russia (1, 57).

Region	Size of population	Thyroid dose (Gy)	
		1 year	Adults
Belarus			
Brest Oblast	1,408,000	0.12	0.026
Vitebsk Oblast	1,410,000	0.007	0.002
Gomel Oblast	1,651,000	0.75	0.15
Grodno Oblast	1,154,000	0.028	0.006
Minsk Oblast	1,587,000	0.016	0.005
Minsk City	1,518,000	0.10	0.018
Mogilev Oblast	1,280,000	0.13	0.031
Ukraine			
Chernihiv Oblast	1,416,000	0.15	0.037
Kyiv Oblast	1,685,000	0.20	0.053
Kyiv City	2,565,000	0.094	0.024
Rivne Oblast	1,164,000	0.15	0.029
Zhytomyr Oblast	1,548,000	0.23	0.060
Russia			
Bryansk Oblast	1,473,000	0.16	0.026
Kaluga Oblasts	1,041,000	0.013	0.002
Orel Oblasts	863,000	0.058	0.009
Tula Oblasts	1,863,000	0.044	0.006

### Thyroid Doses from Pathways Other than Intake of ^131^I

Thyroid doses for most individuals were mainly due to ^131^I intake. However, there were other exposure pathways with typically small contribution to the thyroid dose: (1) intake of short-lived radioiodine and radiotellurium isotopes (^132^I, ^133^I, ^135^I, ^131m^Te, and ^132^Te); (2) external irradiation from gamma-emitting radionuclides deposited on the ground (mainly ^140^Ba+^140^La, ^95^Zr+^90^Nb, and ^132^Te+^132^I shortly after the accident and ^134^Cs and ^137^Cs in the long term); and (3) intake of long-lived ^134^Cs and ^137^Cs. The methods used to estimate thyroid doses from these pathways are described elsewhere (28, 33, 34, 47, 58, 59).

**Table 4** shows the contribution of minor exposure pathways to the individual thyroid doses reconstructed for the subjects of epidemiological studies. The contribution varied from 5 to 8% for subjects of the studies conducted in Belarus and was about 10% for subjects in Russia.

**Table 4:** Contribution of minor exposure pathways to the individual thyroid dose reconstructed for the subjects of epidemiological studies (34, 40, 43, 60).

Pathway	Mean contribution (%) of minor exposure pathways to the individual thyroid dose among the subjects of the study				
	Belarusian–American cohort	Belarusian *in utero* cohort	Case-control study	Case-control study	
	Belarus	Belarus	Belarus	Belarus	Russia
Intake of short-lived radionuclides^a^	2.0	–^b^	2.0	1.6	0.7
External irradiation	4.5	3.6	1.8	3.4	6.3
^137^Cs ingestion	1.5	1.8	1.0	1.3	2.3
All minor exposure pathways	8.0	5.4	4.8	6.3	9.3

^a^Short-lived radioiodine (^132^I, ^133^I, ^135^I) and radiotellurium (^131m^Te and ^132^Te) isotopes.

^b^Not considered.

However, the contribution of minor pathways may be substantial for some individuals. For evacuees from Pripyat-town, which is located near the Chernobyl NPP, the inhalation intake of short-lived radioiodine and radiotellurium isotopes (^132^I, ^133^I, and ^132^Te) contributed about 30% to the total thyroid dose due to inhalation (61). For the IARC coordinated case-control study of thyroid cancer in Belarus and Russia, it was estimated that for 19 out of 1,615 (1.3% of the total) study subjects, the contribution of external irradiation and ingestion of radiocesium isotopes to the thyroid dose was higher than 50% (60). These individuals were relocated from contaminated residents shortly after the accident or did not consume locally produced foodstuffs and, therefore, were exposed to relatively small doses from ^131^I intake in April–June 1986 but received high doses from long-lived sources of exposure in subsequent years.

## Thyroid Doses to The Chernobyl Cleanup Workers

Following the Chernobyl accident, more than 500,000 cleanup workers participated from 26 April 1986 to 31 December 1990 in cleanup activities on the reactor site and in the restricted 30-km zone around the Chernobyl NPP (1). Cleanup workers consisted of different occupational groups, including Chernobyl NPP personnel, nuclear workers, military, construction workers, and support staff, who performed work on decontamination and maintenance at the Chernobyl site, construction and safeguard at various locations (62, 63).

Basically, the cleanup workers received doses due to external irradiation (64–66). However, during the 10-day period of atmospheric releases of radioactivity from destroyed Unit 4, the cleanup workers also were exposed to internal irradiation due to inhalation of ^131^I and short-lived ^132^I, ^133^I, ^135^I, ^131m^Te, and ^132^Te. In addition, cleanup workers, who resided in contaminated areas in Belarus, Ukraine and Russia received thyroid doses due to ^131^I intake with cow’s milk, dairy products and leafy vegetables produced at their places of residence.

### Cleanup Workers With Direct Thyroid Measurements

A group of 594 Chernobyl cleanup workers was measured for ^131^I thyroidal activity between 30 April and 5 May 1986 (67). At the time of the direct thyroid measurement, each person provided information on his or her cleanup activities since 26 April 1986 as well as on stable iodine administration. As measured individuals were the operation personnel of the Chernobyl NPP, only inhalation intake of ^131^I with contaminated air was considered in calculation of the thyroid doses.

**Table 5** shows the distribution of thyroid doses for cleanup workers with direct thyroid measurements. The average thyroid dose among the cleanup workers was 0.18 Gy and the median was 0.11 Gy. About 73% of the cleanup workers received thyroid doses between 0.05 and 0.50 Gy. The highest individual thyroid dose due to ^131^I intake estimated among Chernobyl cleanup workers based on direct thyroid measurement was 4.5 Gy.

**Table 5:** Distribution of the thyroid doses from inhalation of ^131^I for the 594 Chernobyl cleanup workers with direct thyroid measurements (67).

Dose interval (Gy)	N	%	Mean dose in interval (Gy)
<0.02	34	5.7	0.014
0.02–0.049	89	15.0	0.036
0.050–0.099	150	25.2	0.076
0.10–0.199	156	26.3	0.14
0.20–0.499	129	21.7	0.31
0.50–0.999	30	5.1	0.68
≥1.0	6	1.0	1.8
Entire study population	594	100.0	0.18

### Thyroid Doses for the Subjects of Epidemiological Studies

Two case-control thyroid-cancer studies were conducted among Chernobyl cleanup workers: (1) a study nested within cohorts of Belarusian, Russian, and Baltic (Latvia, Lithuania and Estonia) cleanup workers that was coordinated by the IARC and included 530 subjects (19); and (2) a collaborative study of the National Research Center for Radiation Medicine (Kyiv, Ukraine) and the U.S. National Cancer Institute that was nested in a cohort of Ukrainian cleanup workers and included 607 subjects (68). Individual thyroid doses were estimated for the study subjects due to (a) external irradiation during a cleanup mission using the RADRUE method (65), (b) internal irradiation during a cleanup mission due to inhalation of (b) ^131^I and (c) short-lived radioiodine and radiotellurium isotopes as well as from (d) intake of ^131^I during residence (69). A dosimetry questionnaire was administrated to each cleanup worker or his proxy to collect detailed information about (a) the cleanup worker’s routes to and from his or her work place(s) at the Chernobyl site and in the 30-km zone, (b) the details about the cleanup activities, (c) residential history between 26 April and 30 June 1986, and (d) consumption of locally produced foodstuffs (only for Ukrainian cleanup workers). To estimate the uncertainties in doses, *individual stochastic* doses for each considered exposure pathway were calculated using Monte Carlo simulations.

**Table 6** provides the thyroid doses from different exposure pathways reconstructed for the subjects of epidemiological studies among the Chernobyl cleanup workers. The mean thyroid dose from all exposure pathways was 0.19, 0.10, 0.058, and 0.20 Gy for cleanup workers from Belarus, Russia, Baltic States and Ukraine, respectively. The thyroid dose was formed mainly due to external irradiation, except for Belarusian cleanup workers who were, mainly, residents of the 30-km zone and their dose was defined by ^131^I intake during cleanup mission and residence. The thyroid doses during residence were not calculated for Russian and Baltic States cleanup workers as ^131^I deposition densities in their locations of residence were negligible compared to those of Belarusian and Ukrainian cleanup workers.

**Table 6:** Thyroid doses from different exposure pathways reconstructed for subjects of case-control studies of thyroid cancer among Chernobyl cleanup workers (19, 69).

Exposure pathways^a^	Thyroid dose (Gy) for Chernobyl cleanup workers from			
	Belarus	Russia	Baltic States	Ukraine
*External irradiation*				
Arithmetic mean	0.0095	0.10	0.057	0.14
Median	0.0018	0.044	0.039	0.020
Range	~0–0.30	4.8×10^-5^–0.82	7.3×10^-4^–0.26	1.5×10^-5^–3.6
*Inhalation of ^131^I* ^b^				
Arithmetic mean	0.13	–	6.8×10^-4^	0.044
Median	0.024	–	6.8×10^-4^	0.012
Range	3.3×10^-5^–3.2	–	5.2×10^-5^–0.0014	~0–1.7
*Inhalation of short-lived radionuclides*				
Arithmetic mean	–	–	–	0.011
Median	–	–	–	0.0016
Range	–	–	–	~0–0.38
*Intake of ^131^I during residence*				
Arithmetic mean	0.11	–	–	0.042
Median	0.060	–	–	0.0073
Range	2.9×10^-5^–0.73	–	–	~0–3.4
*External irradiation during residence*				
Arithmetic mean	0.0050	–	–	–
Median	0.0011	–	–	–
Range	1.5×10^-5^–0.051	–	–	–
*Total*				
Arithmetic mean^c^	0.19	0.10	0.058	0.20
Median	0.067	0.044	0.040	0.047
Range	1.9×10^-4^–3.3	4.8×10^-5^–0.82	7.3×10^-4^–0.26	1.5×10^-4^–9.0

^a^During the cleanup mission, otherwise indicated.

^b^Both, inhalation and ingestion of ^131^I for cleanup workers from Belarus.

^c^Arithmetic mean, median, and range of thyroid doses are given for the study subjects who were exposed to the specific exposure pathway. Therefore, the arithmetic mean of total dose is not equal to the sum of arithmetic means of components of the dose.

For the IARC-coordinated study, the GSDs of the *individual stochastic* doses due to external irradiation varied from 1.1 to 5.8 with a mean of 1.9, while for the thyroid doses due to ^131^I intake during residence, the GSDs varied from 1.9 to 2.5 with a mean of 2.2 (70). The uncertainties of the *individual stochastic* doses in the Ukrainian-American study were characterized by a mean GSD of 2.0, 1.8, 2.0 and 2.6 for external irradiation, inhalation of ^131^I, inhalation of short-lived radionuclides, and exposure to ^131^I intake during residence, respectively (69).

## Conclusions

This paper considers the radiation exposure to the thyroid after the Chernobyl accident. The most important radiological consequence of the accident was exposure to ^131^I, which led to an increase in the rate of thyroid cancer and other thyroid diseases in the exposed population. The thyroid doses were mainly defined by the consumption of cow’s milk contaminated with ^131^I. Individual thyroid doses due to ^131^I intake varied up to 42 Gy and depended on the age of person, the region where people were exposed, and their cow’s milk consumption habits. In addition to exposure due to ^131^I, the intake of short-lived radioiodine and radiotellurium isotopes, external irradiation, and the intake of long-lived ^134^Cs and ^137^Cs contributed to the thyroid dose of the members of public, typically, not more than 10%.

The mean thyroid dose due to ^131^I inhalation in a group of 594 early Chernobyl cleanup workers was 0.18 Gy. Individual thyroid doses were also reconstructed for 1,137 cleanup workers included in two case-control studies of thyroid cancer. Their thyroid dose from different exposure pathways, i.e., external irradiation, inhalation of ^131^I and short-lived radionuclides during the cleanup mission, and ^131^I intake during residence, varied within a wide range from essential zero to 9 Gy.

## Author Contributions

The author confirms being the sole contributor of this work and has approved it for publication.

## Funding

This work was funded by the Intramural Research Program of the National Cancer Institute (USA), Division of Cancer Epidemiology and Genetics.

## Conflict of Interest

The author declares that the research was conducted in the absence of any commercial or financial relationships that could be construed as a potential conflict of interest.
